# A Dynamic Model and Parameter Identification of High Viscosity Magnetorheological Fluid-Based Energy Absorber with Radial Flow Mode

**DOI:** 10.3390/molecules26227059

**Published:** 2021-11-22

**Authors:** Benyuan Fu, Xianming Zhang, Zhuqiang Li, Ruizhi Shu, Changrong Liao

**Affiliations:** 1 College of Mechanical Engineering, Chongqing University of Technology, Chongqing 400054, China; ruizhishu@cqut.edu.cn; 2 Engineering Research Center for Waste Oil Recovery Technology and Equipment, Ministry of Education, Chongqing Technology and Business University, Chongqing 400067, China; zxm215@126.com (X.Z.); zqli@ctbu.edu.cn (Z.L.); 3 College of Optoelectronic Engineering, Chongqing University, Chongqing 400044, China; crliao@cqu.edu.cn

**Keywords:** dynamic model, magnetorheological energy absorber, impact behavior, parameter identification

## Abstract

The excellent suspension stability of the high-viscosity linear polysiloxane magnetorheological fluid (HVLP MRF) makes it a great controlled medium for magnetorheological energy absorbers (MREAs). In our previous work, the Herschel–Bulkley flow model (HB model) was used to describe the shear-thinning rheological behavior and establish the dynamic model of an HVLP MRF-based MREA with radial flow mode. However, as the established model was implicit, the MREA response time increased and the buffer effect was degraded. To improve the time response characteristics, an explicit dynamic model based on the HB model incorporating minor losses (called the E-HBM model) is proposed in this study. The model parameters were identified based on the HBM model. To verify the E-HBM model, five evaluation parameters for the energy absorption performance of the MREA, that is, peak force, mean force, crush force efficiency, specific energy absorption, and stroke efficiency, were introduced to compare the theoretical results with the experimental results obtained using a high-speed drop tower facility with a mass of 600 kg. Then, the relative error of the crush force efficiency, specific energy absorption, and stroke efficiency was quantitatively and comprehensively analyzed considering the E-HBM model and experimental results. The results indicate that the proposed E-HBM model agrees with the impact behavior of the radial flow mode MREA.

## 1. Introduction

Magnetorheological fluids (MRFs) feature a reversible phase change between fluid and semi-solid states within milliseconds by controlling the applied external magnetic field [1]. Magnetorheological energy absorbers (MREAs), in which the MRF with controllable properties is used as a controlled medium, can adapt their load–stroke profile to varying impact conditions by adjusting the damping force [2,3]. On this basis, MREAs have been extensively investigated for applications in vehicle suspensions [4,5,6], cable-stayed bridges [7,8,9], vibration isolators [10,11], engine mounts [12,13], and washing machines [14]. In these applications, the MRF can maintain a sufficient particle concentration owing to the high frequency of use. This ensures the controllability of MREAs at the time of the energy-absorbing event. However, in applications where MREAs are rarely used, such as energy absorption applications of occupant protection systems [15,16], earthquake mitigation systems [17,18,19], artillery buffer [20,21], and aircraft landing gear [22,23], MRF sedimentation may occur. In case of severe sedimentation, MREAs may fail to provide a controllable damping force when an impact occurs; this significantly degrades their control effect.

A very effective approach to mitigate MRF sedimentation is the use of high-viscosity linear polysiloxane (HVLP) as a new carrier fluid [24]. Xie et al. [25] demonstrated the excellent suspension stability of an HVLP MRF with a carbonyl iron particle volume fraction of 26 vol% and compared it with that of Lord MRF-126CD. After 96 d of measurement, the maximum concentration change for the HVLP MRF was only 1.51%, whereas the Lord MRF-126CD experienced a maximum concentration change of 95.7%. Therefore, the HVLP MRF is suitable for MREAs used in energy absorption applications where MREAs are infrequently used.

As is known, the fast time response characteristic of MREA is essential to control effect [11]. Though the response time of MR material itself is 1–2 ms, the response time of MREA is not as fast as anticipated because other factors (such as compliance of the system, control electronics) affect the total response time of MR devices [26]. Koo et al. [27] focused on the identification of MREA time response. Their results show that the time response depends on several factors: the dwell time of Fe particles in the active zone, the concentration of Fe particles in the carrier fluid, the size of the exciting current in the coil, and the principle of the current control in the electric circuit. In addition to the above-mentioned factors, another important factor affecting the control effect is the operating time of the control system, which controls the output MREA force by adjusting the input current during impact [28]. To achieve the buffering effect, the operation time of the control system should be sufficiently short.

In our prior work [29,30], we proposed a new MREA using the HVLP MRF as a controlled medium to achieve long-term stability. The maximum dynamic range obtained was approximately 1.31 at the drop height of 0.4 m. As HVLP MRF exhibits non-Newtonian fluid characteristics of shearing thinning behavior, the Herschel–Bulkley flow model (HB model) was used to accurately describe its rheological behavior for establishing the implicit dynamic model of an HVLP MRF-based MREA. However, the established implicit dynamic model required an iterative technique to achieve the MREA force during impact, which increased the MREA operating time and degraded the buffering effect. Hence, it is very important to obtain an explicit dynamic model to reduce the operating time of the control system.

Based on the previous analysis, an explicit dynamic model based on the HB model incorporating minor losses (called the E-HBM model) is proposed in this study. According to the design features of the MREA, the E-HBM model force consists of four parts: uncontrollable channel damping force, controllable channel damping force, minor losses damping force, and deformation force of the corrugated tube. The model parameters were identified based on the HBM model. To verify the E-HBM model, five evaluation parameters for the energy absorption performance of MREA (i.e., peak force, mean force [31], crush force efficiency [32], specific energy absorption [33,34], and stroke efficiency [35]) are introduced to compare the theoretical results with the experimental results obtained using a high-speed drop tower facility with a mass of 600 kg. Then, the relative error of the crush force efficiency, specific energy absorption, and stroke efficiency were quantitatively and comprehensively analyzed; these values of the E-HBM model were subsequently compared with the experimental results.

## 2. High-Speed Drop Tower Test

### 2.1. Constitutive Model of HVLP MRF

Unlike conventional MRFs, the HVLP MRF exhibits non-Newtonian fluid characteristics of shearing thinning behavior among its rheological behaviors in the presence of an applied magnetic field. Hence, the HB model was used to describe the rheological behavior [29] as follows:

(1) $$\left\{ \begin{array}{l} \dot{\gamma }=0,\;\;\;\;\;\;\;\;\;\;\;\;\;\;\;\;\;\;\;\;\;\;\;\;\;\;\;\;\;\;\left|\tau \right|<\tau_{y}\;\;\\ \tau =\left(\tau_{y}+K{\left|\dot{\gamma }\right|}^{n}\right)\mathrm{sgn}\left(\dot{\gamma }\right),\;\;\;\;\;\;\;\;\left|\tau \right|>\tau_{y} \end{array}\right.$$

where $\dot{\gamma }$ is the shear rate, $\tau_{y}$ is the dynamic yield stress, K is the post-yield viscosity, and n is a dimensionless behavior index. The HVLP MRF adopted HVLP with a zero-field viscosity of 63 Pa⋅s as the carrier fluid, and magnetic particles with a volume fraction of 26 vol%. Then, the rheological behavior of the HVLP MRF was tested with an MCR-301 rheometer. The HB model parameters were determined using the least-squares regression method under different magnetic fields:

(2) $$\tau_{y}=-0.01023H^{3}+3.51096H^{2}-45.16812H+4888$$

(3) $$K=0.01079H^{2}+1.98112H+44.54$$

(4) $$n=9.95169\times 10^{-10}H^{3}-2.29565\times 10^{-7}H^{2}-6.4643\times 10^{-4}H+0.75$$

### 2.2. MREA Configuration

**Figure 1:**
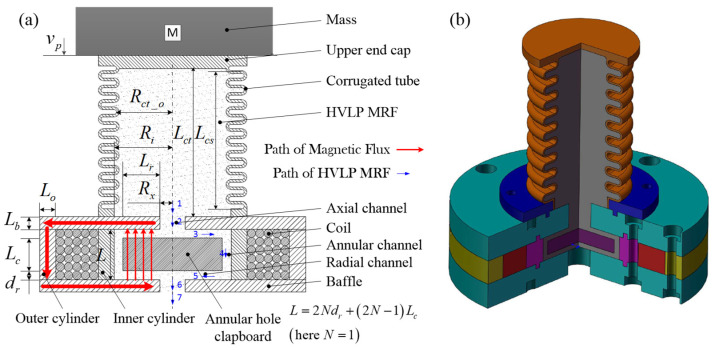
Schematic of an HVLP MRF-based MREA with radial flow mode: (**a**) two-dimensional and (**b**) three-dimensional models. HVLP: high-viscosity linear polysiloxane; MRF: magnetorheological fluid; MREA: magnetorheological energy absorber.

The HVLP MRF-based MREA with radial flow mode is presented in Figure 1; the primary structural dimensions of the MREA are listed in Table 1. The MREA consisted of two parts: a radial MR valve and a corrugated tube assembled on the MR valve. The HVLP MRF was placed in a corrugated tube. During impact, the HVLP MRF was pushed into the MR valve for flow throttling, accompanied by the deformation of the corrugated tube. As the magnetic field generated by coils was perpendicular to the direction of radial flow, the active length of the damping channel was improved. Consequently, the proposed MREA achieved a high magnetic field utilization. The magnetic circuit of the MREA was analyzed using ANSYS. A series of currents from 0.1 A to 3.0 A with intervals of 0.5 A were applied to the coils with 643 turns and coil magnetic wires with a diameter of 0.6 mm; the corresponding magnetic field intensities *H* and magnetic flux density B were obtained. The two magnetic field parameters with respect to the applied current, *I*, are given as follows:

(5) H=151.92⋅I

(6) $$B=0.04262\cdot I^{3}-0.2936\cdot I^{2}+0.83544\cdot I$$

### 2.3. Drop Tower Test Setup

**Figure 2:**
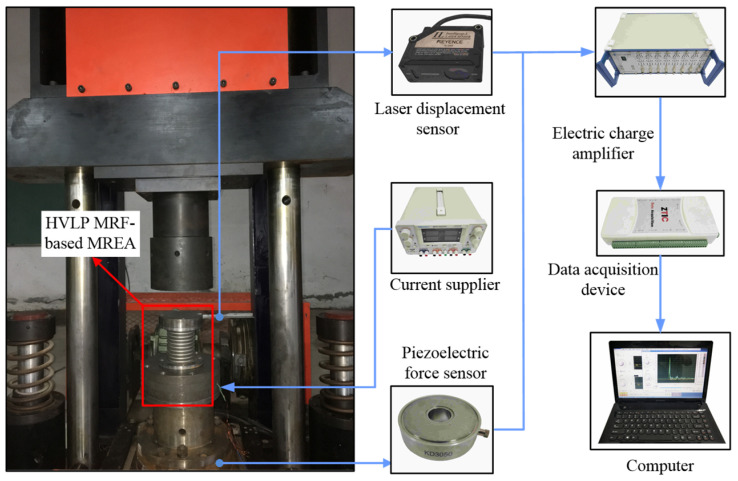
High-mass drop tower test system.

A high-mass drop tower test system was established to evaluate the performance of the HVLP MRF-based MREA under axial impact loading, as shown in Figure 2. The drop tower facility has a drop mass of 600 kg. The MREA prototype was carefully fixed to be concentric with the impact head. During the impact, the excitation current was controlled by a direct current power supply (HSPY-200-05, HSPY, Beijing, China). Impact displacements were obtained using a laser displacement sensor (IL300, KEYENCE, Shanghai, China). The damping forces were obtained using a piezoelectric force sensor (KD3050, KEDONG, Yangzhou, China) below the MREA and an electronic charge amplifier (KD5007G, KEDONG, Yangzhou, China). Then, the damping force and displacement data were transmitted to a computer through a data acquisition device (USB-7646B, ZTIC, Beijing, China). The drop tests were conducted at drop heights of 0.4, 0.7, and 0.9 m. In these tests, the applied current varied discretely with values of 0, 1, 2, and 3 A.

### 2.4. Test Result Analysis

**Figure 3:**
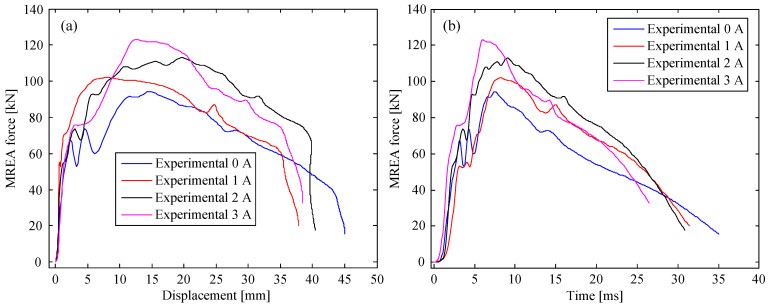
MREA performances at a drop height of 0.4 m: (**a**) force vs. displacement and (**b**) time history of the force.

**Figure 4:**
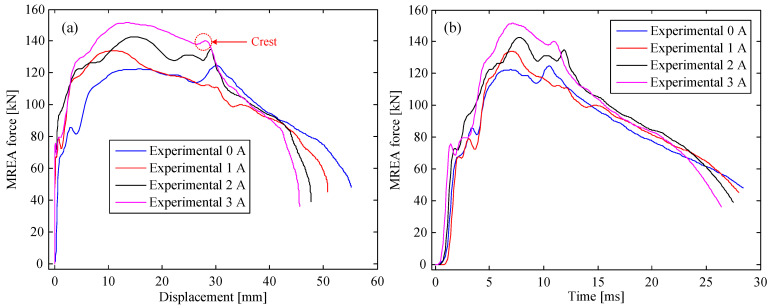
MREA performances at a drop height of 0.7 m: (**a**) force vs. displacement and (**b**) time history of the force.

**Figure 5:**
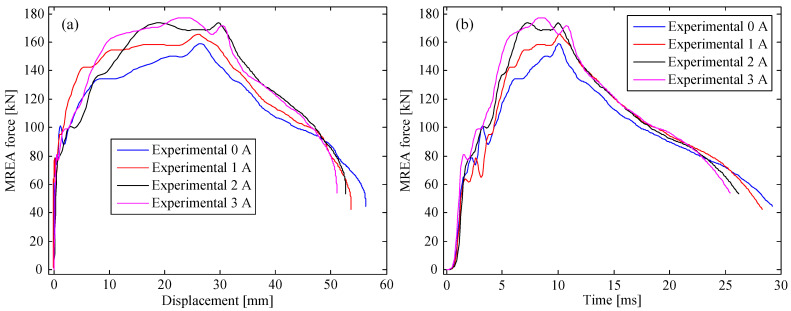
MREA performances at a drop height of 0.9 m: (**a**) force vs. displacement and (**b**) time history of the force.

The experimental results of the MREA for all the cases are plotted in Figure 3, Figure 4 and Figure 5. As observed, the measured MREA force increased with the increasing excitation current, proving the controllability of the HVLP MRF-based MREA. A crest appeared in the MREA force–displacement curve when the displacement was approximately 30 mm, which was a result of the fluid-solid interaction between the HVLP MRF and the corrugated tube. During impact, the HVLP MRF in the corrugated tube was pushed into the MR valve for flow throttling, accompanied by the deformation of the corrugated tube. These two types of energy absorption behavior interacted with each other, producing an extra pressure drop and a large MREA force. Furthermore, the pressure drop in the radial channels increased owing to the increasing applied currents. This was the reason for the decrease in displacement with the increase in applied current, as can be seen in Figure 3a, Figure 4a, and Figure 5a, which reduced the impact time, as seen in Figure 3b, Figure 4b, and Figure 5b.

## 3. MREA Model

During impact, the HVLP MRF was pushed into the MR valve for flow throttling, accompanied by the deformation of the corrugated tube. Thus, the total MREA force can be regarded as a superposition of those of the corrugated tube and MR valve, as follows:

(7) $$F_{MREA}=F_{s}+F_{d}$$

where $F_{MREA}$ is the MREA force, $F_{s}$ is the deformation force of the corrugated tube owing to its deformation, and $F_{d}$ is the total damping force owing to the flow throttling of the HVLP MRF.

### 3.1. Deformation Force

The experimental results show that the maximum displacement range of the MREA was 37–56 mm (related to the impact height and applied current), which was mainly in the elastic and plastic deformation stages of corrugated tubes [36]. However, the elastic deformation stage was only 2–3 mm at the beginning of the impact. Furthermore, the force in the elastic stage was very small and had minimal effect on the impact process. Considering these factors, the deformation force during impact was approximately regarded as in the plastic stage. Thus, the deformation force can be expressed as follows:

(8) $$F_{s}(x)=\frac{\eta_{p}K_{d}G}{EL_{cs}{}^{n_{ct}-1}}\cdot x^{n_{ct}}$$

Here, *K_d_* represents the impact stiffness of the corrugated tube in the elastic stage. *x* is the instantaneous impact displacement. *η_p_* is a compensation coefficient. *E*, *G*, and *n_ct_* are the elastic modulus, strain-strengthening modulus, and plastic strain-strengthening exponent, respectively.

### 3.2. Damping Force Based on the E-HBM Model

According to the HBM dynamic model of the HVLP MRF-based MREA [29,30], the damping force is mainly produced by two types of throttling. The first type is caused by the flow throttling of the HVLP MRF in the corrugated tube, axial channel, and annular and radial channels, which includes the passive damping force and the yield force due to the MR effect. In the corrugated tube, axial channel, and annular channel, the damping forces are not controlled by the magnetic field and have exactly the same mechanical form. These three channels can be collectively called non-controllable channels. By contrast, the damping force in the radial channel is controlled by the magnetic field, which reflects the control effect of the MREA. This channel can be called a controllable channel. The other type is generated by minor losses, which are proportional to the second power of the impact velocity. Although the HBM model can accurately predict the damping force, the iterative technique used during impact increases the operating time of the control system and thus, degrades the buffering effect. This is mainly because the damping force is implicit based on the HBM model. To solve this problem, an explicit dynamic model is proposed, which is an explicit expression of the HBM model (called the E-HBM model). The total damping force based on the E-HBM model can be regarded as a superposition of those of the non-controllable channel, controllable channel, and minor losses, as follows:

(9) $$F_{d}=\left(f_{\tau_{0}}+a\cdot v^{0.75}\right)+\left(f_{\tau }+c\cdot v^{n}\right)+\left(bv^{2}\right)$$

where a and $f_{\tau_{0}}$ are the passive damping coefficient and yield force of the non-controllable channel, respectively, and they are constant. c and $f_{\tau }$ are the passive damping coefficient of the radial channel and yield force due to the MR effect, respectively, which vary with the magnetic field. b is the minor loss damping coefficient.

## 4. Parameter Identification of the E-HBM Model

### 4.1. Model Parameters in the Non-Controllable Channels

From the aforementioned analysis, it can be seen that the corrugated tube, axial channel, and annular channel are non-controllable channels. Therefore, the passive damping coefficient and yield force of these channels can be expressed as follows:

(10) $$a=a_{ct}+a_{x}+a_{n}$$

(11) $$f_{\tau_{0}}=f_{ct}+f_{x}+f_{n}$$

Here, $a_{ct}$, $a_{x}$, and $a_{n}$ are the passive damping coefficients, and $f_{ct}$, $f_{x}$, and $f_{n}$ are the yield forces of the corrugated tube, axial channel, and annular channel, respectively.

#### 4.1.1. Model Parameters in the Corrugated Tube

As the corrugated tube is gradually crushed, its damping length gradually decreases during impact, being a variable related to the impact state. Table 1 shows that the radius of the corrugated tube is considerably larger than that of the other damping channels; thus, its damping force is considerably smaller than that of the other channels. To simplify the calculation, the damping length was estimated as a constant. As observed from Figure 3a and Figure 5a, the maximum displacements of the MREA ranged from 37 to 56 mm. To balance the aforementioned impact conditions, the maximum crush displacement of the corrugated tube was considered as the middle value of the aforesaid maximum displacement range, that is, the maximum crush displacement was approximately $\delta_{\max}=47$ mm. The crush displacement during impact was assumed to be half of the maximum crush displacement, that is, δ=23.5 mm. Therefore, the damping length of the corrugated tube was determined as follows:

(12) $$L_{ct\_impact}=L_{ct}-\delta$$

**Figure 6:**
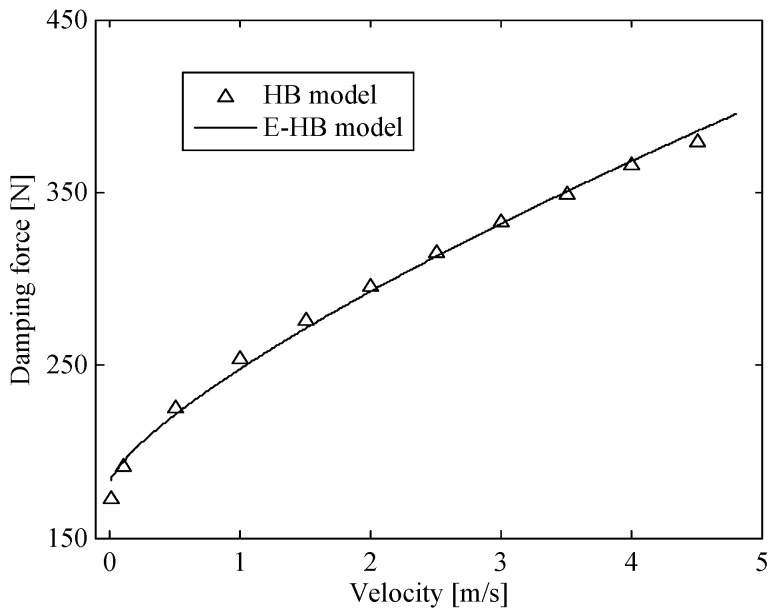
Damping force vs. velocity curve in the corrugated tube.

In the process of model parameter identification, the impact velocity was set as 0.01, 0.1, 0.5–4.5 m/s (interval 0.5 m/s). The variation of the damping force with impact velocity was obtained using the HB model in [29]. Then, the optimized fitting was conducted in accordance with the form of the E-HB model, as shown in Figure 6. As observed, the fitted curve is in good agreement with the HB model curve, with deviations only when the impact velocity was less than 0.01 m/s. The passive damping coefficient and yield force of the corrugated tube were identified as $a_{ct}=79.8$ kg/s and $f_{ct}=220.1$ N.

#### 4.1.2. Model Parameters in Axial and Annular Channels

**Figure 7:**
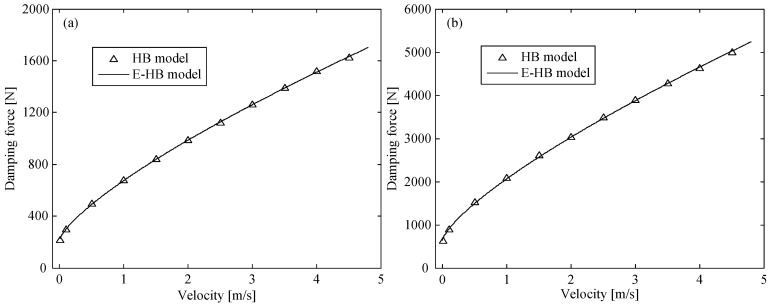
Damping force vs. velocity curve in (**a**) axial and (**b**) annular channels.

Unlike the corrugated tube, the damping lengths of the axial and annular channels are fixed. Thus, the model parameters of the flow channels can be directly identified. During the identification process, the impact velocity was set to 0.01, 0.1, 0.5–4.5 m/s (interval 0.5 m/s). The damping force related to the impact velocity in the axial and annular channels was obtained through the HB model [29]. Then, according to the form of the E-HB model, the damping force vs. velocity curves were optimized, as shown in Figure 7. The fitted curves of the two flow channels were in good agreement with the HB model curve. Therefore, the passive damping coefficient and yield force of the two channels were identified as $a_{x}=458.8$ kg/s, $f_{x}=214.1$ N and $a_{n}=1418.2$ kg/s, $f_{n}=640.6$ N, respectively.

### 4.2. Model Parameters in the Controllable Channel

In the radial channel, the model parameters c, $f_{\tau }$, and n are controlled by the magnetic field, which is controlled by the applied current. The relationship between the parameter n and current I can be determined by substituting Equation (5) into Equation (4), as follows:

(13) $$n=3.4893\times 10^{-3}I^{3}-5.2983\times 10^{-3}I^{2}-9.8206\times 10^{-2}I+0.75$$

**Figure 8:**
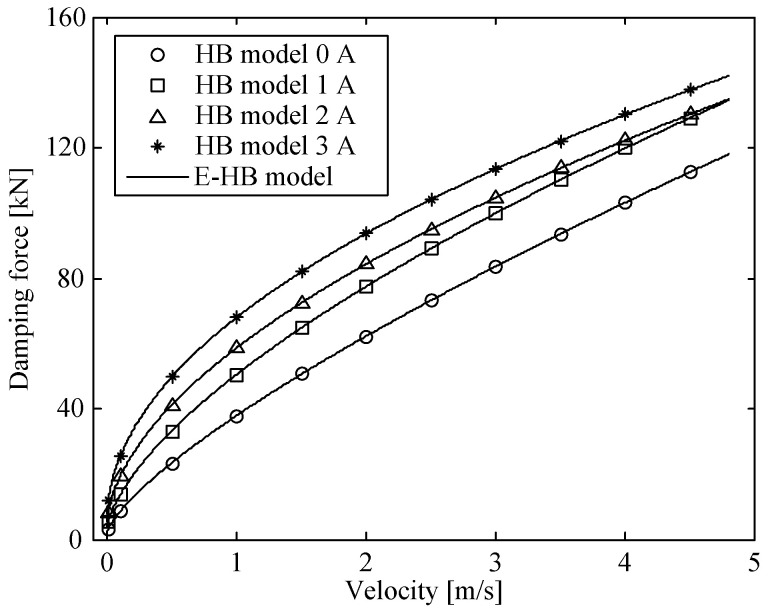
Damping force vs. velocity curves of simplified and HBM models in radial channel.

The relationship between the other two parameters and the current needs to be further identified. According to the aforementioned identification method, the damping forces with respect to the impact velocity in the radial channel at different excitation currents were obtained through the HB model [29]. The damping force vs. velocity curves were optimized in accordance with the form of the E-HB model, as shown in Figure 8. Then, the model parameter values in the radial channel at different excitation currents were obtained, as shown in Table 2.

In addition to the size of the radial channel, the passive damping coefficient c is affected by the HB model parameter K. The combination of Equations (3) and (5) shows that the value of K is ultimately determined by the applied current when the magnetic circuit is not magnetically saturated and can be expressed in the form of the highest second power of the current. Therefore, the relationship between the passive damping coefficient and the applied current can be identified in the same manner. After identification, the passive damping coefficient is expressed as follows:

(14) $$c=-937.5\cdot I^{2}+11430\cdot I+36050$$

The yield force due to the MR effect, $f_{\tau }$, is affected by the HB model parameter $\tau_{y}$. The value of $\tau_{y}$ depends on the applied current, and can be expressed in the form of the highest third power of the current, as shown in Equations (2) and (5). Therefore, the relationship between $f_{\tau }$ and the current can be identified in the same manner. Then, we obtain the yield force in the radial channel as follows:

(15) $$f_{\tau }=-98.02\cdot I^{3}+566.7\cdot I^{2}+372.6\cdot I+2065$$

### 4.3. Damping Coefficient of Minor Losses

For any flow system, there is an additional energy dissipation caused by the so-called minor losses owing to the changes and components of the system. The fluid flow regions in the MR valve are shown in Figure 1. When the fluid flows from region 2 to region 6, the total minor loss pressure drop has five components [29,30]: (1) sudden contraction for flow from regions 2 to 3 and 4 to 5; (2) sudden expansion for flow from regions 3 to 4 and 5 to 6; (3) gradual contraction flow in region 5; (4) gradual expansion flow in region 3; (5) 90°-elbows flow from regions 2 to 3, 3 to 4, 4 to 5, and 5 to 6. The pressure drop induced by these minor losses $\Delta P_{\min}$ is as follows:

(16) $$\Delta P_{\min}=\rho \sum_{i}K_{m\_i}\frac{v_{i}{}^{2}}{2}$$

Here, ρ is the density of the HVLP MRF, $K_{m\_i}$ is the *i*th minor loss coefficient in the flow system, and $v_{i}$ is the corresponding mean fluid velocity associated with this minor loss coefficient. Then, the damping force generated by the minor losses can be obtained by the product of the minor loss pressure drop and the area of the corrugated tube. Finally, the obtained damping force is divided by the quadratic of the impact velocity to obtain the minor loss damping coefficient, b=4831.4 kg/m.

## 5. Evaluation of E-HBM Model

To evaluate the E-HBM model, five parameters for the energy absorption performance of the MREA, that is, peak force, mean force, crush force efficiency, specific energy absorption, and stroke efficiency are introduced and analyzed to compare the E-HBM model with the experimental results. Then, the relative error of the crush force efficiency, specific energy absorption, and stroke efficiency were quantitatively and comprehensively analyzed comparing the theoretical and experimental results.

### 5.1. Peak Force and Mean Force

The MREA peak force, $F_{peak}$, and mean force, $F_{mean}$, are of key interest because they indicate the upper limit of the MREA capability. In addition, plotting the peak/mean force versus applied current for each drop height in the same figure easily demonstrates the adequacy of the E-HBM model in terms of describing the MREA performance. To obtain the mean force of the MREA, the following equation was used:

(17) $$F_{mean}=\frac{E_{MREA}}{\delta }=\frac{\int_{0}^{\delta }F_{MREA}\left(x\right)dx}{\delta }$$

where $F_{MREA}\left(x\right)$, x, δ, and $E_{MREA}$ are the MREA force, corresponding instantaneous displacement, maximum displacement, and total absorbed energy, respectively.

**Figure 9:**
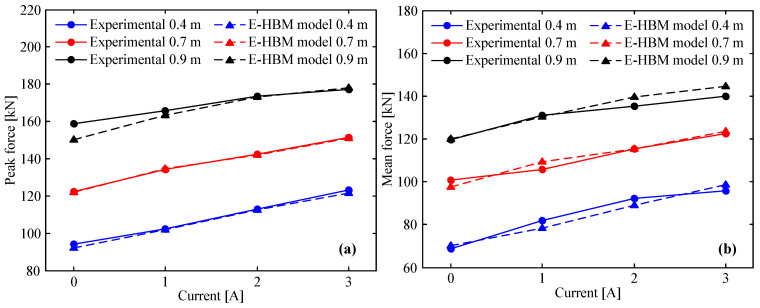
E–HBM model vs. measured data: (**a**) peak force, (**b**) mean force.

Figure 9 shows the peak force and mean force of the MREA vs. the applied current at various drop heights. From the figure, we observe that both the peak force and mean force increase with respect to the applied current. This is because a higher current can increase the yield force of the radial channel owing to the MR effect. Further, Figure 9a shows that the theoretical peak forces of the E-HBM model are in good agreement with the measured data except for the field-off case at a drop height of 0.9 m. As previously analyzed, this error is a result of the fluid–solid interaction between the HVLP MRF and the corrugated tube. From Figure 9b, the tendency of the mean force from the E-HBM model is in good agreement with the measured data as usual. However, there were deviations between the multiple predicted and measured data. This is because the MREA force-displacement curve measured by the experiment was not smooth owing to vibration during impact.

### 5.2. Crush Force Efficiency

The crush force efficiency (*CFE*) is defined as the ratio of the mean force to the peak force. The larger the CFE, the smaller is the difference between the mean force and the peak force, and the stronger the energy absorption capability. The expression for the *CFE* is as follows:

(18) $$CFE=\frac{F_{mean}}{F_{peak}}\times 100\%$$

**Figure 10:**
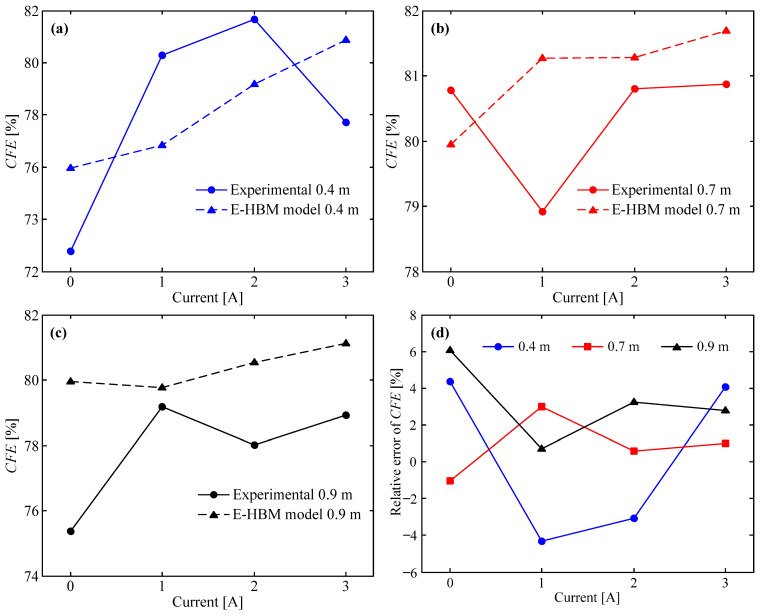
E–HBM model vs. measured data: (**a**) *CFE* at 0.4 m, (**b**) *CFE* at 0.7 m, (**c**) *CFE* at 0.9 m, and (**d**) relative error of *CFE* at different drop heights.

The *CFE* vs. applied current was obtained as shown in Figure 10. For clarity, the results at drop heights of 0.4, 0.7, and 0.9 m are presented in Figure 10a–c, respectively. From these figures, both the predicted and experimental *CFE*s exhibit an upward tendency with increasing current. Experimental *CFE*s at 0.4 m and 3 A, 0.7 m and 1 A, and 0.9 m and 1 A appear to deviate from this tendency, which was caused by the unsmooth MREA force-displacement curve arising from the vibration during impact. In addition, both the experimental and prediction results indicate that the *CFE* was very large. Taking a drop height of 0.4 m (refer to Figure 10a) as an example, the *CFE* of the E-HBM model varied from 75.97 to 80.88%, and the experimental *CFE* varied from 72.78 to 81.68%. Thus, the theoretical result was similar to the experimental result, which indicates that the MREA has a strong energy absorption capability.

To further compare the E-HBM model with the experimental deviation, the relative error of the *CFE* between the prediction and experimental results is introduced as follows:

(19) $$RE_{CFE}=\frac{CFE_{t}-CFE_{e}}{CFE_{e}}\times 100\%$$

where $CFE_{t}$ and $CFE_{e}$ are the crush force efficiencies of the theoretical and experimental results, respectively. The relative error of the *CFE* vs. applied current at various drop heights were acquired, as shown in Figure 10d. It is observed that the error variation ranges with different currents are −4.31–4.38% at 0.4 m, −1.04–2.98% at 0.7 m, and 0.72–6.06% at 0.9 m. In general, the absolute value of $RE_{CFE}$ was relatively small (less than 6.06%), indicating that the E-HBM model has high accuracy for *CFE*.

### 5.3. Specific Energy Absorption

The specific energy absorption (*SEA*) represents the energy absorption per unit mass, which is defined as the ratio of the absorbed energy to the mass of the MREA. The expression is as follows:

(20) $$SEA=\frac{E_{MREA}}{M_{MREA}}$$

where $M_{MREA}$ is the mass of MREA. To evaluate the accuracy of the E-HBM model for *SEA*, the relative error of the SEA between the theoretical and experimental results is introduced as follows:

(21) $$RE_{SEA}=\frac{SEA_{t}-SEA_{e}}{SEA_{e}}\times 100\%$$

where $SEA_{t}$ and $SEA_{e}$ are the specific energy absorptions of the theoretical and experimental results, respectively.

**Figure 11:**
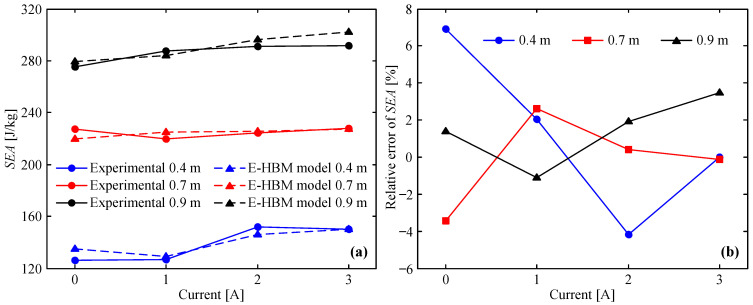
E–HBM model vs. measured data at different drop heights: (**a**) *SEA*, (**b**) relative error of *SEA*.

The *SEA* from the E-HBM model and measured data vs. applied current is plotted in Figure 11a. As can be seen in this figure, the changing tendency of the theoretical *SEA* using the E-HBM model is in good agreement with the measured data as usual. However, the theoretical *SEA* is evidently larger than the experimental value under two impact conditions, that is, the theoretical value is 8.7 J/kg and 10.1 J/kg larger at 0.4 m and 0 A and 0.9 m and 3 A, respectively. Moreover, the theoretical *SEA* is evidently 6.3 J/kg and 7.8 J/kg smaller at 0.4 m and 2 A and 0.7 m and 0 A than the experimental value, respectively. This demonstrates that a deviation still exists between the theoretical and measured results. The relative error between the theoretical *SEA* and experimental results, shown in Figure 11b, was relatively large under the four aforementioned impact conditions. Its value was 6.92% at 0.4 m and 0 A, 3.48% at 0.9 m and 3 A, −4.18% at 0.4 m and 2 A, and −3.43% at 0.7 m and 0 A, respectively. Overall, the absolute value of $RE_{SEA}$ between the theoretical and experimental results was smaller than 6.92%, indicating that the E-HBM model can provide a high-accuracy prediction for *SEA*.

### 5.4. Stroke Efficiency

Stroke efficiency (*SE*) is defined as the ratio of the maximum displacement to the corrugated segment length of the corrugated tube. The expression is as follows:

(22) $$SE=\frac{\delta }{L_{cs}}\times 100\%$$

To evaluate the prediction accuracy of the *SE* using the E-HBM model, the relative error of the *SE* is introduced to quantitatively compare the theoretical and experimental results. Its expression is as follows:

(23) $$RE_{SE}=\frac{SE_{t}-SE_{e}}{SE_{e}}\times 100\%$$

where $SE_{t}$ and $SE_{e}$ are the stroke efficiencies of the theoretical and experimental results, respectively.

**Figure 12:**
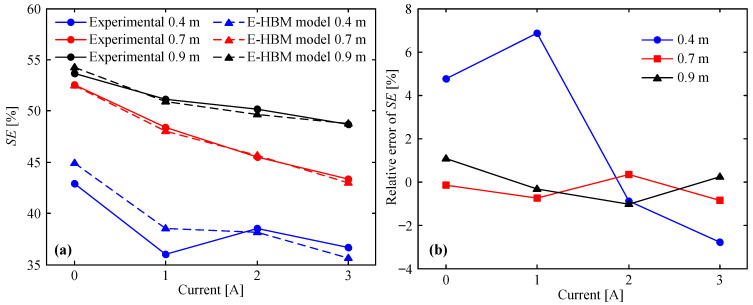
E–HBM model vs. measured data at different drop heights: (**a**) *SE*, (**b**) relative error of *SE*.

Figure 12a shows the theoretical and measured *SE* vs. applied current at different drop heights. As can be seen in this figure, both the theoretical and measured *SE*s decreased as the applied current increased; this was because a larger applied current increased the MREA force and reduced its maximum displacement at the same drop height. Furthermore, the field-off *SE* at 0.9 m was slightly larger than that at 0.7 m, although the drop height increased by 0.2 m. This phenomenon is attributed to the fact that only small extra stroke efficiency was required because the field-off MREA force at 0.9 m was considerably larger than that at 0.7 m (refer to Figure 4 and Figure 5). In addition, the theoretical *SE* with 0 A and 1 A at 0.4 m were larger than the measured data. The main reason for this is that the yield force in the controllable channel given by Equation (15) was slightly smaller than the actual situation. The relative error of the SE between the theoretical and experimental results is shown in Figure 12b. It is observed that the relative error was 4.75% at 0.4 m and 0 A, 6.86% at 0.4 m and 1 A, and −2.80% at 0.4 m and 3 A. The relative error under the other impact conditions was lower than 2%. Therefore, the E-HBM model is very effective for predicting *SE*.

## 6. Conclusions

In this study, an explicit E-HBM dynamic model of the HVLP MRF-based MREA with radial flow mode was proposed. According to the MREA structure, the total damping force based on the E-HBM model can be regarded as a superposition of those of the non-controllable channel, controllable channel, and minor losses. The relevant HBM model parameters were identified based on the damping forces of the HB model. To verify the E-HBM model, five evaluation parameters, that is, peak force, mean force, crush force efficiency, specific energy absorption, and stroke efficiency, were analyzed to compare the theoretical results with the experimental results obtained using a high-speed drop tower facility with a mass of 600 kg. Overall, the five theoretical evaluation parameters were in good agreement with the experimental results. The absolute values of $RE_{CFE}$, $RE_{SEA}$, and $RE_{SE}$ were less than 6.06% (at 0.9 m and 0 A), 6.92% (at 0.4 m and 0 A), and 6.86% (at 0.4 m and 1 A), respectively. The main reason for this error is that the experimental MREA force–displacement curve was not sufficiently smooth owing to the vibration during impact. Hence, the E-HBM model can provide high-accuracy prediction for the five parameters analyzed, demonstrating that the E-HBM model can effectively predict the dynamic behavior and performance of the HVLP MRF-based MREA under impact conditions.

## Figures and Tables

**Table 1:** Dimensions of HVLP MRF-based MREA with radial flow mode.

Parameter	Value
Inner radius of the inner cylinder, *R_i_*	48 mm
Radius of the axial channel, *R_x_*	14 mm
Length of the cylinder, *L*	24 mm
Thickness of the clapboard, *L_c_*	20 mm
Single-stage radial damping length, *L_r_*	30 mm
Thickness of the baffle, *L_b_*	25 mm
Thickness of the outer cylinder, *L_o_*	20 mm
Length of corrugated tube, *L_ct_*	135 mm
Corrugated segment length of corrugated tube, *L_cs_*	105 mm
Effective MR valve gap width of the radial channels, *d_r_*	2 mm
Number range of overall stages in the MR valve, *N*	1
Coil turn numbers, *Turn*	643 Turn
Outer radius of straight segment, *R_ct_o_*	34 mm

**Table 2:** Model parameters values in the radial channel at different currents.

Current [A]	Passive Damping Coefficient [kg/s]	Yield Force [N]
0	35,742	2065
1	47,472	2906.3
2	54,240	4293
3	62,220	5637

## Data Availability

All relevant data are included in the article.

## Sample Availability

Samples of the compounds are not available from the authors.
